# Nonacarbonyl-1κ^3^ *C*,2κ^3^ *C*,3κ^3^ *C*-μ-bis­(diphenyl­arsino)methane-1:2κ^2^ *As*:*As*’-[tris­(2-chloro­eth­yl) phosphite-3κ*P*]-*triangulo*-triruthenium(0)

**DOI:** 10.1107/S1600536810026267

**Published:** 2010-07-14

**Authors:** Omar bin Shawkataly, Imthyaz Ahmed Khan, Siti Syaida Sirat, Chin Sing Yeap, Hoong-Kun Fun

**Affiliations:** a Chemical Sciences Programme, School of Distance Education, Universiti Sains Malaysia, 11800 USM, Penang, Malaysia; b X-ray Crystallography Unit, School of Physics, Universiti Sains Malaysia, 11800 USM, Penang, Malaysia

## Abstract

In the title *triangulo*-triruthenium(0) compound, [Ru_3_(C_25_H_22_As_2_)(C_6_H_12_Cl_3_O_3_P)(CO)_9_], the bis­(diphenyl­arsino)methane ligand bridges an Ru—Ru bond and the monodentate phosphine ligand bonds to the third Ru atom. Both the arsine and phosphine ligands are equatorial with respect to the Ru_3_ triangle. In addition, each Ru atom carries one equatorial and two axial terminal carbonyl ligands. In the crystal packing, the mol­ecules are linked by inter­molecular C—H⋯O hydrogen bonds into a three-dimensional framework. Weak inter­molecular C—H⋯π inter­actions further stabilize the crystal structure.

## Related literature

For general background to *triangulo*-triruthenium derivatives, see: Bruce *et al.* (1985 ▶, 1988*a* ▶,*b* ▶). For related structures, see: Shawkataly *et al.* (1998 ▶, 2004 ▶, 2010 ▶). For the synthesis of μ-bis­(diphenyl­arsino)methane­deca­carbonyl­triruthenium(0), see: Bruce *et al.* (1983 ▶). For the stability of the temperature controller used for the data collection, see: Cosier & Glazer (1986 ▶).
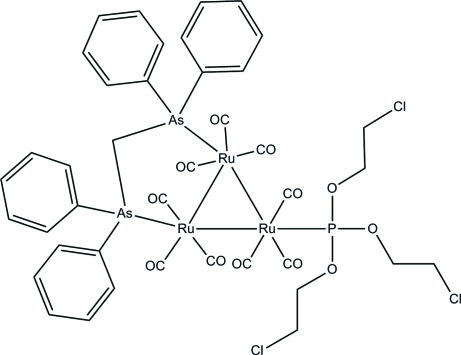


## Experimental

### 

#### Crystal data

[Ru_3_(C_25_H_22_As_2_)(C_6_H_12_Cl_3_O_3_P)(CO)_9_]
*M* *_r_* = 1297.04
Orthorhombic, 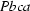

*a* = 14.9105 (5) Å
*b* = 21.3468 (7) Å
*c* = 28.7377 (9) Å
*V* = 9147.0 (5) Å^3^
*Z* = 8
Mo *K*α radiation
μ = 2.68 mm^−1^
*T* = 100 K
0.47 × 0.18 × 0.09 mm

#### Data collection

Bruker SMART APEXII CCD area-detector diffractometer
Absorption correction: multi-scan (*SADABS*; Bruker, 2009 ▶) *T*_min_ = 0.365, *T*_max_ = 0.798
55092 measured reflections
10492 independent reflections
8686 reflections with *I* > 2σ(*I*)
*R*_int_ = 0.047

#### Refinement

*R*[*F* ^2^ > 2σ(*F* ^2^)] = 0.080
*wR*(*F* ^2^) = 0.184
*S* = 1.28
10492 reflections
532 parameters
H-atom parameters constrained
Δρ_max_ = 1.40 e Å^−3^
Δρ_min_ = −2.28 e Å^−3^

### 

Data collection: *APEX2* (Bruker, 2009 ▶); cell refinement: *SAINT* (Bruker, 2009 ▶); data reduction: *SAINT*; program(s) used to solve structure: *SHELXTL* (Sheldrick, 2008 ▶); program(s) used to refine structure: *SHELXTL*; molecular graphics: *SHELXTL*; software used to prepare material for publication: *SHELXTL* and *PLATON* (Spek, 2009 ▶).

## Supplementary Material

Crystal structure: contains datablocks global, I. DOI: 10.1107/S1600536810026267/sj5029sup1.cif

Structure factors: contains datablocks I. DOI: 10.1107/S1600536810026267/sj5029Isup2.hkl

Additional supplementary materials:  crystallographic information; 3D view; checkCIF report

## Figures and Tables

**Table 1:** Hydrogen-bond geometry (Å, °) *Cg*1 and *Cg*2 are the centroids of the C1–C6 and C20–C25 benzene rings, respectively.

*D*—H⋯*A*	*D*—H	H⋯*A*	*D*⋯*A*	*D*—H⋯*A*
C19—H19*A*⋯O6^i^	0.93	2.57	3.275 (14)	133
C27—H27*B*⋯O9^ii^	0.97	2.47	3.231 (15)	135
C30—H30*A*⋯O5^iii^	0.97	2.56	3.297 (15)	133
C4—H4*A*⋯*Cg*1^iv^	0.93	2.77	3.468 (11)	133
C9—H9*A*⋯*Cg*2^v^	0.93	2.89	3.684 (12)	144
C18—H18*A*⋯*Cg*1^vi^	0.93	2.71	3.505 (12)	145
C24—H24*A*⋯*Cg*2^vii^	0.93	2.69	3.473 (11)	142

Symmetry codes: (i) 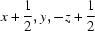; (ii) 
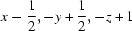
; (iii) 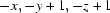; (iv) 
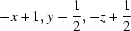
; (v) 
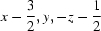
; (vi) 
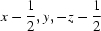
; (vii) 
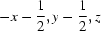
.

**Fig. 1.**
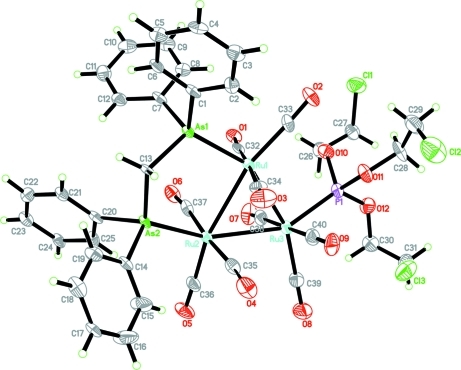
The molecular structure of the title compound with 50% probability ellipsoids for non-H atoms.

**Fig. 2.**
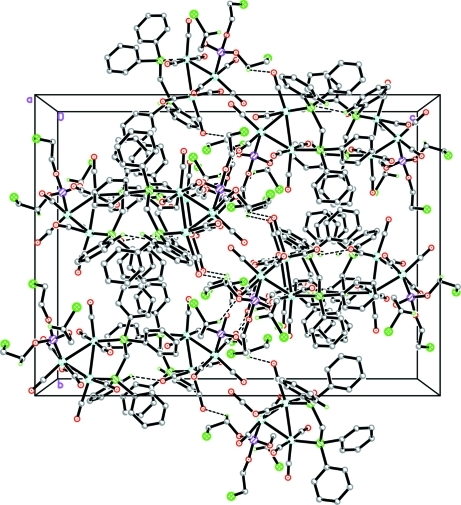
The crystal packing of the title compound, viewed down the a axis, showing the molecules linked into a 3-D framework. Hydrogen atoms not involved in the hydrogen-bonding (dashed lines) have been omitted for clarity.

## supplementary crystallographic information

### Comment

*Triangulo*-triruthenium clusters are known for their interesting structural variations and related catalytic activity. A large number of substituted derivatives, Ru_3_(CO)_12-n_*L*_n_ (*L*= group 15 ligand) have been reported (Bruce, *et al.*, 1988*a,b*; Bruce *et al.*, 1985). As part of our study of the substitution of transition metal-carbonyl clusters with mixed-ligand complexes, we have published several structures of *triangulo*-triruthenium-carbonyl clusters containing mixed P/As and P/Sb ligands (Shawkataly *et al.*, 1998, 2004, 2010). Herein we report the synthesis and structure of the title compound.

The bond lengths and angles of title compound (Fig. 1) are comparable to those found in a related structure (Shawkataly *et al.*, 2010). The bis(diphenylarsino)methane ligand bridges the Ru1—Ru2 bond and the monodentate phosphine ligand bonds to the Ru3 atom. Both the phosphine and arsine ligands are equatorial with respect to the Ru_3_ triangle. Additionally, each Ru atom carries one equatorial and two axial terminal carbonyl ligands. The dihedral angles between the two benzene rings (C1–C6/C7–C12 and C14–C19/C20–C25) are 85.4 (5) and 87.3 (5)° for the two diphenylarsino groups respectively.

In the crystal packing (Fig. 2), the molecules are linked by intermolecular C19—H19A···O6, C27—H27B···O9 and C30—H30A···O5 hydrogen bonds into a three-dimensional framework. Weak intermolecular C—H···π interactions further stabilize the crystal structure (Table 1).

### Experimental

All manipulations were performed under a dry, oxygen-free dinitrogen atmosphere using standard Schlenk techniques. All solvents were dried over sodium and distilled from sodium benzophenone ketyl under nitrogen. Tris(2-chloroethyl)phosphite (Aldrich) was used as received and µ-bis(diphenylarsino)methanedecacarbonyltriruthenium(0) (Bruce *et al.*, 1983) was prepared by a reported procedure. The title compound was obtained by refluxing equimolar quantities of Ru_3_(CO)_10_(µ-Ph_2_AsCH_2_AsPh_2_) and tris(2-chloroethyl)phosphite in hexane under nitrogen atmosphere. Crystals suitable for X-ray diffraction were grown by slow solvent / solvent diffusion of CH_3_OH into CH_2_Cl_2_.

### Refinement

All hydrogen atoms were positioned geometrically and refined using a riding model with C—H = 0.93 Å and *U*_iso_(H) = 1.2*U*_eq_(C). The same U^ij^ parameters were used for the atoms C17/C1/C20/C7. The maximum and minimum residual electron density peaks of 1.40 and -2.28 e Å^-3^, respectively, were located 0.79 Å and 1.42 Å from the RU1 and C36 atoms, repectively.

### Crystal data

**Table:**

[Ru_3_(C_25_H_22_As_2_)(C_6_H_12_Cl_3_O_3_P)(CO)_9_]	*F*(000) = 5072
*M**_r_* = 1297.04	*D*_x_ = 1.884 Mg m^−^^3^
Orthorhombic, *P**b**c**a*	Mo *K*α radiation, λ = 0.71073 Å
Hall symbol: -P 2ac 2ab	Cell parameters from 9974 reflections
*a* = 14.9105 (5) Å	θ = 2.2–30.0°
*b* = 21.3468 (7) Å	µ = 2.68 mm^−^^1^
*c* = 28.7377 (9) Å	*T* = 100 K
*V* = 9147.0 (5) Å^3^	Block, red
*Z* = 8	0.47 × 0.18 × 0.09 mm

### Data collection

**Table:**

Bruker SMART APEXII CCD area-detector diffractometer	10492 independent reflections
Radiation source: fine-focus sealed tube	8686 reflections with *I* > 2σ(*I*)
graphite	*R*_int_ = 0.047
φ and ω scans	θ_max_ = 27.5°, θ_min_ = 2.0°
Absorption correction: multi-scan (*SADABS*; Bruker, 2009)	*h* = −19→19
*T*_min_ = 0.365, *T*_max_ = 0.798	*k* = −24→27
55092 measured reflections	*l* = −37→28

### Refinement

**Table:**

Refinement on *F*^2^	Primary atom site location: structure-invariant direct methods
Least-squares matrix: full	Secondary atom site location: difference Fourier map
*R*[*F*^2^ > 2σ(*F*^2^)] = 0.080	Hydrogen site location: inferred from neighbouring sites
*wR*(*F*^2^) = 0.184	H-atom parameters constrained
*S* = 1.28	*w* = 1/[σ^2^(*F*_o_^2^) + 274.9171*P*] where *P* = (*F*_o_^2^ + 2*F*_c_^2^)/3
10492 reflections	(Δ/σ)_max_ < 0.001
532 parameters	Δρ_max_ = 1.40 e Å^−^^3^
0 restraints	Δρ_min_ = −2.28 e Å^−^^3^

### Special details

**Table:**

Experimental. The crystal was placed in the cold stream of an Oxford Cryosystems Cobra open-flow nitrogen cryostat (Cosier & Glazer, 1986) operating at 100.0 (1) K.
Geometry. All e.s.d.'s (except the e.s.d. in the dihedral angle between two l.s. planes) are estimated using the full covariance matrix. The cell e.s.d.'s are taken into account individually in the estimation of e.s.d.'s in distances, angles and torsion angles; correlations between e.s.d.'s in cell parameters are only used when they are defined by crystal symmetry. An approximate (isotropic) treatment of cell e.s.d.'s is used for estimating e.s.d.'s involving l.s. planes.
Refinement. Refinement of *F*^2^ against ALL reflections. The weighted *R*-factor *wR* and goodness of fit *S* are based on *F*^2^, conventional *R*-factors *R* are based on *F*, with *F* set to zero for negative *F*^2^. The threshold expression of *F*^2^ > σ(*F*^2^) is used only for calculating *R*-factors(gt) *etc*. and is not relevant to the choice of reflections for refinement. *R*-factors based on *F*^2^ are statistically about twice as large as those based on *F*, and *R*- factors based on ALL data will be even larger.

### Fractional atomic coordinates and isotropic or equivalent isotropic displacement parameters (Å^2^)

**Table:**

	*x*	*y*	*z*	*U*_iso_*/*U*_eq_	
Ru1	0.21161 (5)	0.32808 (4)	0.36287 (3)	0.01629 (17)	
Ru2	0.24882 (5)	0.45825 (4)	0.37688 (3)	0.01781 (17)	
Ru3	0.13426 (6)	0.39328 (4)	0.43926 (3)	0.01984 (18)	
As1	0.29465 (6)	0.32478 (4)	0.29063 (3)	0.0149 (2)	
As2	0.36319 (6)	0.46579 (4)	0.31669 (3)	0.0157 (2)	
Cl1	−0.1760 (2)	0.20128 (17)	0.38798 (12)	0.0469 (8)	
Cl2	0.1764 (3)	0.1315 (2)	0.51607 (17)	0.0651 (11)	
Cl3	0.0908 (3)	0.33271 (19)	0.58014 (12)	0.0515 (9)	
P1	0.03663 (18)	0.31463 (13)	0.45706 (10)	0.0220 (5)	
O1	0.0321 (5)	0.3570 (3)	0.3155 (3)	0.0248 (16)	
O2	0.1464 (6)	0.1935 (4)	0.3698 (3)	0.0343 (19)	
O3	0.3930 (6)	0.3016 (5)	0.4095 (3)	0.042 (2)	
O4	0.3881 (6)	0.4318 (4)	0.4543 (3)	0.043 (2)	
O5	0.2262 (6)	0.5934 (4)	0.4072 (3)	0.042 (2)	
O6	0.1079 (5)	0.4855 (4)	0.3018 (3)	0.0314 (18)	
O7	−0.0137 (6)	0.4633 (4)	0.3861 (3)	0.039 (2)	
O8	0.1295 (7)	0.4908 (4)	0.5165 (3)	0.047 (2)	
O9	0.2772 (6)	0.3235 (4)	0.4958 (3)	0.038 (2)	
O10	−0.0249 (5)	0.2907 (4)	0.4148 (3)	0.0279 (17)	
O11	0.0830 (5)	0.2504 (4)	0.4713 (3)	0.0301 (18)	
O12	−0.0405 (5)	0.3227 (4)	0.4958 (3)	0.0320 (18)	
C1	0.3636 (7)	0.2498 (5)	0.2759 (4)	0.0209 (10)	
C2	0.3736 (7)	0.2020 (5)	0.3078 (4)	0.023 (2)	
H2A	0.3465	0.2052	0.3369	0.028*	
C3	0.4249 (7)	0.1481 (5)	0.2966 (4)	0.026 (2)	
H3A	0.4326	0.1159	0.3181	0.032*	
C4	0.4632 (7)	0.1445 (5)	0.2528 (4)	0.025 (2)	
H4A	0.4964	0.1093	0.2449	0.030*	
C5	0.4535 (7)	0.1918 (5)	0.2209 (4)	0.024 (2)	
H5A	0.4807	0.1887	0.1918	0.028*	
C6	0.4033 (7)	0.2442 (5)	0.2319 (4)	0.022 (2)	
H6A	0.3958	0.2759	0.2100	0.026*	
C7	0.2258 (6)	0.3321 (5)	0.2337 (4)	0.0209 (10)	
C8	0.1547 (7)	0.2896 (5)	0.2294 (4)	0.024 (2)	
H8A	0.1413	0.2628	0.2539	0.028*	
C9	0.1037 (7)	0.2870 (5)	0.1886 (4)	0.029 (2)	
H9A	0.0571	0.2583	0.1856	0.035*	
C10	0.1238 (7)	0.3274 (6)	0.1532 (4)	0.029 (2)	
H10A	0.0896	0.3264	0.1262	0.035*	
C11	0.1936 (8)	0.3698 (5)	0.1567 (4)	0.030 (2)	
H11A	0.2059	0.3969	0.1323	0.036*	
C12	0.2454 (8)	0.3717 (5)	0.1970 (4)	0.024 (2)	
H12A	0.2931	0.3995	0.1993	0.029*	
C13	0.3935 (6)	0.3858 (4)	0.2876 (4)	0.0155 (18)	
H13A	0.4457	0.3683	0.3030	0.019*	
H13B	0.4090	0.3930	0.2552	0.019*	
C14	0.4795 (7)	0.4968 (5)	0.3366 (4)	0.021 (2)	
C15	0.4857 (9)	0.5300 (7)	0.3778 (5)	0.046 (4)	
H15A	0.4354	0.5359	0.3965	0.055*	
C16	0.5688 (9)	0.5547 (8)	0.3910 (5)	0.055 (5)	
H16A	0.5735	0.5770	0.4187	0.066*	
C17	0.6439 (7)	0.5466 (4)	0.3639 (4)	0.0209 (10)	
H17A	0.6989	0.5628	0.3732	0.025*	
C18	0.6363 (8)	0.5143 (5)	0.3227 (4)	0.031 (3)	
H18A	0.6865	0.5098	0.3038	0.037*	
C19	0.5559 (7)	0.4884 (5)	0.3089 (4)	0.024 (2)	
H19A	0.5524	0.4655	0.2814	0.029*	
C20	0.3436 (7)	0.5224 (4)	0.2645 (4)	0.0209 (10)	
C21	0.3840 (6)	0.5139 (5)	0.2209 (4)	0.019 (2)	
H21A	0.4181	0.4783	0.2151	0.023*	
C22	0.3731 (6)	0.5589 (5)	0.1862 (4)	0.022 (2)	
H22A	0.3978	0.5519	0.1570	0.026*	
C23	0.3264 (7)	0.6135 (4)	0.1943 (4)	0.022 (2)	
H23A	0.3219	0.6442	0.1715	0.026*	
C24	0.2863 (7)	0.6216 (5)	0.2374 (4)	0.022 (2)	
H24A	0.2530	0.6576	0.2431	0.027*	
C25	0.2949 (7)	0.5764 (5)	0.2724 (4)	0.027 (2)	
H25A	0.2677	0.5828	0.3011	0.033*	
C26	−0.1120 (8)	0.3167 (6)	0.4048 (4)	0.034 (3)	
H26A	−0.1172	0.3248	0.3717	0.041*	
H26B	−0.1189	0.3563	0.4211	0.041*	
C27	−0.1841 (8)	0.2726 (6)	0.4197 (4)	0.032 (3)	
H27A	−0.2423	0.2915	0.4144	0.038*	
H27B	−0.1784	0.2641	0.4528	0.038*	
C28	0.0311 (9)	0.1978 (6)	0.4858 (5)	0.042 (3)	
H28A	−0.0234	0.1956	0.4674	0.050*	
H28B	0.0142	0.2029	0.5182	0.050*	
C29	0.0825 (10)	0.1385 (6)	0.4802 (5)	0.045 (3)	
H29A	0.1019	0.1352	0.4481	0.054*	
H29B	0.0426	0.1036	0.4864	0.054*	
C30	−0.0498 (8)	0.3739 (6)	0.5280 (4)	0.032 (3)	
H30A	−0.1113	0.3888	0.5279	0.039*	
H30B	−0.0113	0.4082	0.5185	0.039*	
C31	−0.0249 (10)	0.3533 (7)	0.5760 (4)	0.042 (3)	
H31A	−0.0613	0.3174	0.5846	0.050*	
H31B	−0.0378	0.3868	0.5978	0.050*	
C32	0.0983 (7)	0.3500 (4)	0.3339 (4)	0.021 (2)	
C33	0.1725 (7)	0.2440 (5)	0.3684 (4)	0.024 (2)	
C34	0.3240 (8)	0.3135 (5)	0.3939 (4)	0.027 (2)	
C35	0.3336 (7)	0.4384 (6)	0.4253 (4)	0.030 (3)	
C36	0.2369 (7)	0.5416 (5)	0.3969 (4)	0.029 (2)	
C37	0.1579 (7)	0.4715 (5)	0.3307 (4)	0.024 (2)	
C38	0.0446 (8)	0.4367 (5)	0.4036 (4)	0.027 (2)	
C39	0.1298 (8)	0.4546 (5)	0.4874 (4)	0.029 (2)	
C40	0.2281 (7)	0.3489 (6)	0.4725 (4)	0.029 (2)	

### Atomic displacement parameters (Å^2^)

**Table:**

	*U*^11^	*U*^22^	*U*^33^	*U*^12^	*U*^13^	*U*^23^
Ru1	0.0141 (3)	0.0125 (3)	0.0223 (4)	0.0005 (3)	0.0010 (3)	0.0020 (3)
Ru2	0.0153 (3)	0.0145 (4)	0.0236 (4)	−0.0007 (3)	0.0036 (3)	−0.0003 (3)
Ru3	0.0196 (4)	0.0174 (4)	0.0225 (4)	0.0016 (3)	0.0041 (3)	0.0026 (3)
As1	0.0125 (4)	0.0089 (4)	0.0231 (5)	0.0008 (3)	0.0007 (4)	0.0007 (4)
As2	0.0133 (4)	0.0085 (4)	0.0251 (5)	0.0000 (4)	0.0029 (4)	0.0003 (4)
Cl1	0.0447 (18)	0.0480 (19)	0.0480 (19)	−0.0177 (16)	0.0021 (15)	−0.0110 (16)
Cl2	0.059 (2)	0.050 (2)	0.086 (3)	0.0054 (19)	−0.023 (2)	0.012 (2)
Cl3	0.060 (2)	0.060 (2)	0.0340 (16)	0.0100 (19)	−0.0097 (15)	−0.0030 (16)
P1	0.0208 (13)	0.0198 (13)	0.0254 (13)	0.0000 (10)	0.0029 (10)	0.0038 (10)
O1	0.017 (4)	0.023 (4)	0.034 (4)	0.000 (3)	−0.004 (3)	−0.001 (3)
O2	0.033 (4)	0.018 (4)	0.052 (5)	−0.002 (3)	0.003 (4)	0.007 (4)
O3	0.027 (4)	0.050 (6)	0.049 (5)	0.009 (4)	−0.017 (4)	−0.011 (4)
O4	0.031 (5)	0.043 (5)	0.054 (6)	−0.009 (4)	−0.021 (4)	0.015 (4)
O5	0.041 (5)	0.024 (5)	0.062 (6)	−0.008 (4)	0.023 (4)	−0.019 (4)
O6	0.024 (4)	0.026 (4)	0.044 (5)	−0.001 (3)	−0.007 (4)	0.010 (4)
O7	0.031 (4)	0.044 (5)	0.043 (5)	0.017 (4)	0.006 (4)	0.014 (4)
O8	0.060 (6)	0.035 (5)	0.045 (5)	−0.004 (5)	0.011 (5)	−0.011 (4)
O9	0.041 (5)	0.042 (5)	0.033 (4)	0.005 (4)	−0.006 (4)	0.018 (4)
O10	0.020 (4)	0.027 (4)	0.036 (4)	−0.004 (3)	−0.001 (3)	−0.003 (3)
O11	0.024 (4)	0.023 (4)	0.043 (5)	−0.002 (3)	0.007 (3)	0.007 (4)
O12	0.028 (4)	0.038 (5)	0.030 (4)	−0.003 (4)	0.010 (3)	−0.003 (4)
C1	0.017 (2)	0.011 (2)	0.035 (3)	−0.0042 (18)	0.001 (2)	−0.002 (2)
C2	0.017 (5)	0.027 (5)	0.026 (5)	−0.004 (4)	−0.003 (4)	0.003 (4)
C3	0.029 (6)	0.020 (5)	0.031 (6)	0.003 (4)	−0.011 (5)	0.003 (4)
C4	0.025 (5)	0.016 (5)	0.034 (6)	0.002 (4)	−0.005 (4)	−0.008 (4)
C5	0.017 (5)	0.021 (5)	0.033 (6)	0.004 (4)	0.001 (4)	−0.005 (4)
C6	0.025 (5)	0.010 (4)	0.030 (5)	−0.002 (4)	0.003 (4)	0.001 (4)
C7	0.017 (2)	0.011 (2)	0.035 (3)	−0.0042 (18)	0.001 (2)	−0.002 (2)
C8	0.023 (5)	0.022 (5)	0.027 (5)	0.000 (4)	−0.001 (4)	−0.003 (4)
C9	0.016 (5)	0.030 (6)	0.040 (6)	0.000 (4)	0.001 (5)	−0.009 (5)
C10	0.023 (5)	0.032 (6)	0.033 (6)	0.004 (5)	−0.009 (4)	−0.008 (5)
C11	0.037 (6)	0.024 (6)	0.029 (6)	0.003 (5)	−0.003 (5)	0.006 (5)
C12	0.025 (5)	0.017 (5)	0.031 (5)	0.001 (4)	0.002 (5)	−0.003 (4)
C13	0.007 (4)	0.006 (4)	0.034 (5)	0.001 (3)	0.003 (4)	−0.003 (4)
C14	0.018 (5)	0.013 (5)	0.031 (5)	−0.005 (4)	0.001 (4)	0.001 (4)
C15	0.028 (6)	0.061 (9)	0.049 (8)	−0.024 (6)	0.012 (6)	−0.026 (7)
C16	0.038 (7)	0.081 (11)	0.047 (8)	−0.029 (8)	0.012 (6)	−0.038 (8)
C17	0.017 (2)	0.011 (2)	0.035 (3)	−0.0042 (18)	0.001 (2)	−0.002 (2)
C18	0.020 (5)	0.023 (5)	0.050 (7)	0.005 (4)	0.002 (5)	0.007 (5)
C19	0.023 (5)	0.014 (5)	0.036 (6)	0.005 (4)	0.000 (4)	−0.004 (4)
C20	0.017 (2)	0.011 (2)	0.035 (3)	−0.0042 (18)	0.001 (2)	−0.002 (2)
C21	0.009 (4)	0.019 (5)	0.031 (5)	−0.002 (4)	0.002 (4)	−0.001 (4)
C22	0.012 (4)	0.029 (5)	0.024 (5)	0.000 (4)	0.002 (4)	−0.001 (4)
C23	0.022 (5)	0.010 (4)	0.034 (6)	0.005 (4)	−0.003 (4)	0.005 (4)
C24	0.021 (5)	0.012 (5)	0.035 (6)	0.000 (4)	0.009 (4)	0.001 (4)
C25	0.017 (5)	0.029 (6)	0.036 (6)	0.000 (4)	0.009 (4)	0.005 (5)
C26	0.032 (6)	0.033 (7)	0.037 (7)	−0.002 (5)	−0.005 (5)	0.003 (5)
C27	0.027 (6)	0.030 (6)	0.038 (6)	−0.003 (5)	0.001 (5)	0.002 (5)
C28	0.044 (7)	0.023 (6)	0.057 (8)	−0.011 (6)	0.003 (6)	0.017 (6)
C29	0.054 (9)	0.028 (7)	0.053 (8)	−0.005 (6)	−0.005 (7)	0.005 (6)
C30	0.028 (6)	0.030 (6)	0.039 (7)	0.005 (5)	0.009 (5)	−0.004 (5)
C31	0.052 (8)	0.042 (7)	0.031 (6)	−0.010 (6)	0.016 (6)	−0.004 (6)
C32	0.029 (6)	0.008 (4)	0.026 (5)	−0.002 (4)	0.004 (4)	−0.001 (4)
C33	0.021 (5)	0.021 (5)	0.031 (6)	0.001 (4)	0.002 (4)	0.010 (4)
C34	0.032 (6)	0.022 (5)	0.026 (5)	0.003 (5)	−0.003 (5)	−0.008 (4)
C35	0.016 (5)	0.038 (6)	0.036 (6)	−0.018 (5)	0.003 (5)	0.000 (5)
C36	0.024 (5)	0.026 (6)	0.036 (6)	−0.008 (5)	0.007 (5)	0.000 (5)
C37	0.019 (5)	0.018 (5)	0.036 (6)	0.002 (4)	0.007 (4)	0.007 (4)
C38	0.027 (6)	0.025 (6)	0.031 (6)	0.000 (5)	0.013 (5)	0.000 (5)
C39	0.037 (6)	0.018 (5)	0.031 (6)	0.007 (5)	0.010 (5)	−0.002 (5)
C40	0.026 (6)	0.031 (6)	0.029 (6)	−0.006 (5)	0.000 (5)	0.004 (5)

### Geometric parameters (Å, °)

**Table:**

Ru1—C33	1.894 (11)	C7—C12	1.381 (14)
Ru1—C34	1.924 (11)	C7—C8	1.400 (14)
Ru1—C32	1.941 (11)	C8—C9	1.397 (15)
Ru1—As1	2.4182 (12)	C8—H8A	0.9300
Ru1—Ru3	2.8437 (11)	C9—C10	1.367 (17)
Ru1—Ru2	2.8621 (11)	C9—H9A	0.9300
Ru2—C36	1.878 (12)	C10—C11	1.383 (16)
Ru2—C37	1.917 (11)	C10—H10A	0.9300
Ru2—C35	1.928 (12)	C11—C12	1.392 (15)
Ru2—As2	2.4341 (12)	C11—H11A	0.9300
Ru2—Ru3	2.8382 (11)	C12—H12A	0.9300
Ru3—C39	1.904 (11)	C13—H13A	0.9700
Ru3—C38	1.923 (12)	C13—H13B	0.9700
Ru3—C40	1.942 (12)	C14—C15	1.383 (16)
Ru3—P1	2.280 (3)	C14—C19	1.401 (15)
As1—C7	1.938 (11)	C15—C16	1.399 (17)
As1—C1	1.948 (10)	C15—H15A	0.9300
As1—C13	1.969 (9)	C16—C17	1.374 (16)
As2—C14	1.943 (10)	C16—H16A	0.9300
As2—C20	1.949 (11)	C17—C18	1.374 (16)
As2—C13	1.955 (9)	C17—H17A	0.9300
Cl1—C27	1.779 (12)	C18—C19	1.379 (15)
Cl2—C29	1.744 (15)	C18—H18A	0.9300
Cl3—C31	1.784 (15)	C19—H19A	0.9300
P1—O11	1.589 (8)	C20—C25	1.383 (14)
P1—O10	1.605 (8)	C20—C21	1.401 (14)
P1—O12	1.610 (8)	C21—C22	1.394 (14)
O1—C32	1.130 (13)	C21—H21A	0.9300
O2—C33	1.147 (13)	C22—C23	1.378 (14)
O3—C34	1.151 (14)	C22—H22A	0.9300
O4—C35	1.172 (14)	C23—C24	1.385 (15)
O5—C36	1.157 (14)	C23—H23A	0.9300
O6—C37	1.155 (13)	C24—C25	1.398 (15)
O7—C38	1.153 (14)	C24—H24A	0.9300
O8—C39	1.139 (14)	C25—H25A	0.9300
O9—C40	1.131 (14)	C26—C27	1.492 (16)
O10—C26	1.442 (14)	C26—H26A	0.9700
O11—C28	1.427 (13)	C26—H26B	0.9700
O12—C30	1.440 (14)	C27—H27A	0.9700
C1—C2	1.378 (14)	C27—H27B	0.9700
C1—C6	1.402 (14)	C28—C29	1.487 (18)
C2—C3	1.420 (15)	C28—H28A	0.9700
C2—H2A	0.9300	C28—H28B	0.9700
C3—C4	1.382 (16)	C29—H29A	0.9700
C3—H3A	0.9300	C29—H29B	0.9700
C4—C5	1.373 (15)	C30—C31	1.493 (18)
C4—H4A	0.9300	C30—H30A	0.9700
C5—C6	1.382 (14)	C30—H30B	0.9700
C5—H5A	0.9300	C31—H31A	0.9700
C6—H6A	0.9300	C31—H31B	0.9700
			
C33—Ru1—C34	94.3 (5)	C11—C10—H10A	119.2
C33—Ru1—C32	89.8 (4)	C10—C11—C12	119.8 (11)
C34—Ru1—C32	175.0 (4)	C10—C11—H11A	120.1
C33—Ru1—As1	101.7 (3)	C12—C11—H11A	120.1
C34—Ru1—As1	86.9 (3)	C7—C12—C11	120.0 (10)
C32—Ru1—As1	94.8 (3)	C7—C12—H12A	120.0
C33—Ru1—Ru3	105.9 (3)	C11—C12—H12A	120.0
C34—Ru1—Ru3	94.3 (3)	As2—C13—As1	112.7 (4)
C32—Ru1—Ru3	81.9 (3)	As2—C13—H13A	109.1
As1—Ru1—Ru3	152.20 (4)	As1—C13—H13A	109.1
C33—Ru1—Ru2	165.5 (3)	As2—C13—H13B	109.1
C34—Ru1—Ru2	85.6 (3)	As1—C13—H13B	109.1
C32—Ru1—Ru2	89.7 (3)	H13A—C13—H13B	107.8
As1—Ru1—Ru2	92.85 (4)	C15—C14—C19	119.9 (10)
Ru3—Ru1—Ru2	59.66 (3)	C15—C14—As2	119.1 (8)
C36—Ru2—C37	90.3 (5)	C19—C14—As2	120.9 (8)
C36—Ru2—C35	92.9 (5)	C14—C15—C16	118.8 (11)
C37—Ru2—C35	174.6 (4)	C14—C15—H15A	120.6
C36—Ru2—As2	102.8 (3)	C16—C15—H15A	120.6
C37—Ru2—As2	89.7 (3)	C17—C16—C15	121.5 (12)
C35—Ru2—As2	93.9 (3)	C17—C16—H16A	119.3
C36—Ru2—Ru3	102.3 (3)	C15—C16—H16A	119.3
C37—Ru2—Ru3	94.8 (3)	C16—C17—C18	119.0 (10)
C35—Ru2—Ru3	80.3 (3)	C16—C17—H17A	120.5
As2—Ru2—Ru3	154.52 (4)	C18—C17—H17A	120.5
C36—Ru2—Ru1	160.8 (3)	C17—C18—C19	121.4 (11)
C37—Ru2—Ru1	84.8 (3)	C17—C18—H18A	119.3
C35—Ru2—Ru1	90.9 (4)	C19—C18—H18A	119.3
As2—Ru2—Ru1	95.74 (4)	C18—C19—C14	119.4 (10)
Ru3—Ru2—Ru1	59.85 (3)	C18—C19—H19A	120.3
C39—Ru3—C38	91.8 (5)	C14—C19—H19A	120.3
C39—Ru3—C40	90.2 (5)	C25—C20—C21	118.7 (10)
C38—Ru3—C40	177.2 (5)	C25—C20—As2	118.0 (8)
C39—Ru3—P1	108.7 (4)	C21—C20—As2	122.9 (7)
C38—Ru3—P1	91.7 (3)	C22—C21—C20	120.0 (9)
C40—Ru3—P1	89.5 (3)	C22—C21—H21A	120.0
C39—Ru3—Ru2	98.3 (3)	C20—C21—H21A	120.0
C38—Ru3—Ru2	81.2 (3)	C23—C22—C21	121.4 (10)
C40—Ru3—Ru2	96.6 (3)	C23—C22—H22A	119.3
P1—Ru3—Ru2	152.34 (8)	C21—C22—H22A	119.3
C39—Ru3—Ru1	155.6 (4)	C22—C23—C24	118.3 (9)
C38—Ru3—Ru1	96.1 (3)	C22—C23—H23A	120.8
C40—Ru3—Ru1	81.3 (3)	C24—C23—H23A	120.8
P1—Ru3—Ru1	94.11 (8)	C23—C24—C25	121.2 (9)
Ru2—Ru3—Ru1	60.49 (3)	C23—C24—H24A	119.4
C7—As1—C1	99.3 (4)	C25—C24—H24A	119.4
C7—As1—C13	107.8 (4)	C20—C25—C24	120.3 (10)
C1—As1—C13	98.0 (4)	C20—C25—H25A	119.8
C7—As1—Ru1	116.8 (3)	C24—C25—H25A	119.8
C1—As1—Ru1	118.7 (3)	O10—C26—C27	110.4 (10)
C13—As1—Ru1	113.7 (3)	O10—C26—H26A	109.6
C14—As2—C20	98.6 (4)	C27—C26—H26A	109.6
C14—As2—C13	102.5 (4)	O10—C26—H26B	109.6
C20—As2—C13	104.3 (4)	C27—C26—H26B	109.6
C14—As2—Ru2	116.0 (3)	H26A—C26—H26B	108.1
C20—As2—Ru2	118.9 (3)	C26—C27—Cl1	110.2 (9)
C13—As2—Ru2	114.1 (3)	C26—C27—H27A	109.6
O11—P1—O10	99.7 (4)	Cl1—C27—H27A	109.6
O11—P1—O12	103.0 (4)	C26—C27—H27B	109.6
O10—P1—O12	98.6 (4)	Cl1—C27—H27B	109.6
O11—P1—Ru3	114.5 (3)	H27A—C27—H27B	108.1
O10—P1—Ru3	115.4 (3)	O11—C28—C29	111.0 (11)
O12—P1—Ru3	122.2 (3)	O11—C28—H28A	109.4
C26—O10—P1	122.8 (7)	C29—C28—H28A	109.4
C28—O11—P1	121.2 (8)	O11—C28—H28B	109.4
C30—O12—P1	126.5 (7)	C29—C28—H28B	109.4
C2—C1—C6	119.3 (10)	H28A—C28—H28B	108.0
C2—C1—As1	121.4 (8)	C28—C29—Cl2	115.1 (10)
C6—C1—As1	119.3 (8)	C28—C29—H29A	108.5
C1—C2—C3	120.5 (10)	Cl2—C29—H29A	108.5
C1—C2—H2A	119.7	C28—C29—H29B	108.5
C3—C2—H2A	119.7	Cl2—C29—H29B	108.5
C4—C3—C2	118.2 (10)	H29A—C29—H29B	107.5
C4—C3—H3A	120.9	O12—C30—C31	110.2 (10)
C2—C3—H3A	120.9	O12—C30—H30A	109.6
C5—C4—C3	121.6 (10)	C31—C30—H30A	109.6
C5—C4—H4A	119.2	O12—C30—H30B	109.6
C3—C4—H4A	119.2	C31—C30—H30B	109.6
C4—C5—C6	119.9 (10)	H30A—C30—H30B	108.1
C4—C5—H5A	120.1	C30—C31—Cl3	112.0 (9)
C6—C5—H5A	120.1	C30—C31—H31A	109.2
C5—C6—C1	120.3 (10)	Cl3—C31—H31A	109.2
C5—C6—H6A	119.8	C30—C31—H31B	109.2
C1—C6—H6A	119.8	Cl3—C31—H31B	109.2
C12—C7—C8	119.3 (10)	H31A—C31—H31B	107.9
C12—C7—As1	125.5 (8)	O1—C32—Ru1	173.4 (9)
C8—C7—As1	115.1 (8)	O2—C33—Ru1	176.6 (10)
C9—C8—C7	120.8 (10)	O3—C34—Ru1	174.5 (10)
C9—C8—H8A	119.6	O4—C35—Ru2	173.8 (10)
C7—C8—H8A	119.6	O5—C36—Ru2	176.3 (11)
C10—C9—C8	118.6 (10)	O6—C37—Ru2	172.6 (9)
C10—C9—H9A	120.7	O7—C38—Ru3	173.4 (10)
C8—C9—H9A	120.7	O8—C39—Ru3	178.1 (12)
C9—C10—C11	121.6 (10)	O9—C40—Ru3	172.9 (10)
C9—C10—H10A	119.2		
			
C33—Ru1—Ru2—C36	−30.3 (17)	C36—Ru2—As2—C13	179.9 (5)
C34—Ru1—Ru2—C36	−120.6 (12)	C37—Ru2—As2—C13	−89.9 (4)
C32—Ru1—Ru2—C36	57.8 (12)	C35—Ru2—As2—C13	86.1 (5)
As1—Ru1—Ru2—C36	152.7 (11)	Ru3—Ru2—As2—C13	10.5 (3)
Ru3—Ru1—Ru2—C36	−23.0 (11)	Ru1—Ru2—As2—C13	−5.2 (3)
C33—Ru1—Ru2—C37	−105.9 (13)	C39—Ru3—P1—O11	−109.6 (5)
C34—Ru1—Ru2—C37	163.7 (5)	C38—Ru3—P1—O11	157.9 (5)
C32—Ru1—Ru2—C37	−17.8 (4)	C40—Ru3—P1—O11	−19.6 (5)
As1—Ru1—Ru2—C37	77.0 (3)	Ru2—Ru3—P1—O11	83.8 (4)
Ru3—Ru1—Ru2—C37	−98.6 (3)	Ru1—Ru3—P1—O11	61.7 (4)
C33—Ru1—Ru2—C35	70.9 (13)	C39—Ru3—P1—O10	135.5 (5)
C34—Ru1—Ru2—C35	−19.4 (5)	C38—Ru3—P1—O10	43.0 (5)
C32—Ru1—Ru2—C35	159.1 (4)	C40—Ru3—P1—O10	−134.5 (5)
As1—Ru1—Ru2—C35	−106.1 (3)	Ru2—Ru3—P1—O10	−31.1 (4)
Ru3—Ru1—Ru2—C35	78.2 (3)	Ru1—Ru3—P1—O10	−53.2 (3)
C33—Ru1—Ru2—As2	164.9 (13)	C39—Ru3—P1—O12	15.9 (5)
C34—Ru1—Ru2—As2	74.6 (3)	C38—Ru3—P1—O12	−76.6 (5)
C32—Ru1—Ru2—As2	−106.9 (3)	C40—Ru3—P1—O12	105.9 (5)
As1—Ru1—Ru2—As2	−12.09 (4)	Ru2—Ru3—P1—O12	−150.8 (4)
Ru3—Ru1—Ru2—As2	172.24 (4)	Ru1—Ru3—P1—O12	−172.9 (4)
C33—Ru1—Ru2—Ru3	−7.3 (13)	O11—P1—O10—C26	145.8 (8)
C34—Ru1—Ru2—Ru3	−97.6 (3)	O12—P1—O10—C26	40.9 (9)
C32—Ru1—Ru2—Ru3	80.8 (3)	Ru3—P1—O10—C26	−91.0 (8)
As1—Ru1—Ru2—Ru3	175.68 (4)	O10—P1—O11—C28	−59.6 (10)
C36—Ru2—Ru3—C39	−20.3 (5)	O12—P1—O11—C28	41.7 (10)
C37—Ru2—Ru3—C39	−111.6 (5)	Ru3—P1—O11—C28	176.6 (9)
C35—Ru2—Ru3—C39	70.5 (5)	O11—P1—O12—C30	120.5 (9)
As2—Ru2—Ru3—C39	149.0 (4)	O10—P1—O12—C30	−137.4 (9)
Ru1—Ru2—Ru3—C39	167.2 (4)	Ru3—P1—O12—C30	−9.9 (11)
C36—Ru2—Ru3—C38	70.2 (5)	C7—As1—C1—C2	−134.8 (8)
C37—Ru2—Ru3—C38	−21.1 (5)	C13—As1—C1—C2	115.6 (9)
C35—Ru2—Ru3—C38	161.1 (5)	Ru1—As1—C1—C2	−7.1 (10)
As2—Ru2—Ru3—C38	−120.4 (4)	C7—As1—C1—C6	44.8 (9)
Ru1—Ru2—Ru3—C38	−102.2 (3)	C13—As1—C1—C6	−64.8 (9)
C36—Ru2—Ru3—C40	−111.5 (5)	Ru1—As1—C1—C6	172.5 (7)
C37—Ru2—Ru3—C40	157.2 (5)	C6—C1—C2—C3	1.1 (15)
C35—Ru2—Ru3—C40	−20.6 (5)	As1—C1—C2—C3	−179.3 (8)
As2—Ru2—Ru3—C40	57.9 (4)	C1—C2—C3—C4	−0.8 (15)
Ru1—Ru2—Ru3—C40	76.1 (4)	C2—C3—C4—C5	0.8 (16)
C36—Ru2—Ru3—P1	146.9 (4)	C3—C4—C5—C6	−0.9 (16)
C37—Ru2—Ru3—P1	55.6 (4)	C4—C5—C6—C1	1.2 (16)
C35—Ru2—Ru3—P1	−122.3 (4)	C2—C1—C6—C5	−1.3 (15)
As2—Ru2—Ru3—P1	−43.8 (2)	As1—C1—C6—C5	179.1 (8)
Ru1—Ru2—Ru3—P1	−25.54 (17)	C1—As1—C7—C12	−99.4 (9)
C36—Ru2—Ru3—Ru1	172.4 (4)	C13—As1—C7—C12	2.1 (10)
C37—Ru2—Ru3—Ru1	81.1 (3)	Ru1—As1—C7—C12	131.7 (8)
C35—Ru2—Ru3—Ru1	−96.7 (4)	C1—As1—C7—C8	75.7 (8)
As2—Ru2—Ru3—Ru1	−18.21 (10)	C13—As1—C7—C8	177.3 (7)
C33—Ru1—Ru3—C39	146.1 (9)	Ru1—As1—C7—C8	−53.2 (8)
C34—Ru1—Ru3—C39	50.3 (9)	C12—C7—C8—C9	−0.1 (16)
C32—Ru1—Ru3—C39	−126.4 (9)	As1—C7—C8—C9	−175.5 (8)
As1—Ru1—Ru3—C39	−41.3 (8)	C7—C8—C9—C10	−1.0 (16)
Ru2—Ru1—Ru3—C39	−32.0 (8)	C8—C9—C10—C11	0.9 (17)
C33—Ru1—Ru3—C38	−105.7 (5)	C9—C10—C11—C12	0.2 (18)
C34—Ru1—Ru3—C38	158.6 (5)	C8—C7—C12—C11	1.2 (16)
C32—Ru1—Ru3—C38	−18.2 (4)	As1—C7—C12—C11	176.1 (8)
As1—Ru1—Ru3—C38	66.9 (3)	C10—C11—C12—C7	−1.2 (17)
Ru2—Ru1—Ru3—C38	76.2 (3)	C14—As2—C13—As1	152.2 (5)
C33—Ru1—Ru3—C40	75.4 (5)	C20—As2—C13—As1	−105.4 (5)
C34—Ru1—Ru3—C40	−20.4 (5)	Ru2—As2—C13—As1	25.9 (6)
C32—Ru1—Ru3—C40	162.9 (4)	C7—As1—C13—As2	93.3 (6)
As1—Ru1—Ru3—C40	−112.0 (3)	C1—As1—C13—As2	−164.1 (5)
Ru2—Ru1—Ru3—C40	−102.7 (3)	Ru1—As1—C13—As2	−37.9 (6)
C33—Ru1—Ru3—P1	−13.5 (3)	C20—As2—C14—C15	108.7 (11)
C34—Ru1—Ru3—P1	−109.2 (4)	C13—As2—C14—C15	−144.4 (10)
C32—Ru1—Ru3—P1	74.0 (3)	Ru2—As2—C14—C15	−19.4 (11)
As1—Ru1—Ru3—P1	159.13 (11)	C20—As2—C14—C19	−68.2 (9)
Ru2—Ru1—Ru3—P1	168.42 (8)	C13—As2—C14—C19	38.6 (9)
C33—Ru1—Ru3—Ru2	178.1 (3)	Ru2—As2—C14—C19	163.6 (7)
C34—Ru1—Ru3—Ru2	82.3 (3)	C19—C14—C15—C16	0(2)
C32—Ru1—Ru3—Ru2	−94.4 (3)	As2—C14—C15—C16	−177.1 (12)
As1—Ru1—Ru3—Ru2	−9.29 (9)	C14—C15—C16—C17	0(3)
C33—Ru1—As1—C7	83.0 (5)	C15—C16—C17—C18	1(2)
C34—Ru1—As1—C7	176.8 (5)	C16—C17—C18—C19	−1.9 (18)
C32—Ru1—As1—C7	−7.9 (4)	C17—C18—C19—C14	2.0 (16)
Ru3—Ru1—As1—C7	−89.8 (3)	C15—C14—C19—C18	−1.0 (17)
Ru2—Ru1—As1—C7	−97.8 (3)	As2—C14—C19—C18	176.0 (8)
C33—Ru1—As1—C1	−36.0 (5)	C14—As2—C20—C25	−94.4 (9)
C34—Ru1—As1—C1	57.8 (5)	C13—As2—C20—C25	160.3 (8)
C32—Ru1—As1—C1	−126.8 (4)	Ru2—As2—C20—C25	31.8 (9)
Ru3—Ru1—As1—C1	151.3 (3)	C14—As2—C20—C21	79.0 (9)
Ru2—Ru1—As1—C1	143.2 (3)	C13—As2—C20—C21	−26.3 (9)
C33—Ru1—As1—C13	−150.4 (4)	Ru2—As2—C20—C21	−154.8 (7)
C34—Ru1—As1—C13	−56.6 (4)	C25—C20—C21—C22	−1.4 (14)
C32—Ru1—As1—C13	118.8 (4)	As2—C20—C21—C22	−174.7 (7)
Ru3—Ru1—As1—C13	36.9 (3)	C20—C21—C22—C23	3.0 (15)
Ru2—Ru1—As1—C13	28.8 (3)	C21—C22—C23—C24	−3.2 (15)
C36—Ru2—As2—C14	61.0 (5)	C22—C23—C24—C25	1.8 (16)
C37—Ru2—As2—C14	151.2 (5)	C21—C20—C25—C24	0.1 (15)
C35—Ru2—As2—C14	−32.8 (5)	As2—C20—C25—C24	173.8 (8)
Ru3—Ru2—As2—C14	−108.3 (3)	C23—C24—C25—C20	−0.3 (17)
Ru1—Ru2—As2—C14	−124.1 (3)	P1—O10—C26—C27	−105.3 (10)
C36—Ru2—As2—C20	−56.4 (5)	O10—C26—C27—Cl1	−62.3 (12)
C37—Ru2—As2—C20	33.8 (5)	P1—O11—C28—C29	158.8 (9)
C35—Ru2—As2—C20	−150.2 (5)	O11—C28—C29—Cl2	65.8 (15)
Ru3—Ru2—As2—C20	134.3 (3)	P1—O12—C30—C31	−107.2 (11)
Ru1—Ru2—As2—C20	118.5 (3)	O12—C30—C31—Cl3	65.1 (12)

### Hydrogen-bond geometry (Å, °)

**Table:**

Cg1 and Cg2 are the centroids of the C1–C6 and C20–C25 benzene rings, respectively.

**Table:**

*D*—H···*A*	*D*—H	H···*A*	*D*···*A*	*D*—H···*A*
C19—H19A···O6^i^	0.93	2.57	3.275 (14)	133
C27—H27B···O9^ii^	0.97	2.47	3.231 (15)	135
C30—H30A···O5^iii^	0.97	2.56	3.297 (15)	133
C4—H4A···Cg1^iv^	0.93	2.77	3.468 (11)	133
C9—H9A···Cg2^v^	0.93	2.89	3.684 (12)	144
C18—H18A···Cg1^vi^	0.93	2.71	3.505 (12)	145
C24—H24A···Cg2^vii^	0.93	2.69	3.473 (11)	142

Symmetry codes: (i) *x*+1/2, *y*, −*z*+1/2; (ii) *x*−1/2, −*y*+1/2, −*z*+1; (iii) −*x*, −*y*+1, −*z*+1; (iv) −*x*+1, *y*−1/2, −*z*+1/2; (v) *x*−3/2, *y*, −*z*−1/2; (vi) *x*−1/2, *y*, −*z*−1/2; (vii) −*x*−1/2, *y*−1/2, *z*.
